# A multicenter study on the mental health of Brazilian adolescent mothers, 2024

**DOI:** 10.1590/S2237-96222025v34e20240226.en

**Published:** 2025-09-08

**Authors:** Amanda Ferreira de Carvalho, Daniela Dal Forno Kinalski Guaranha, Bruna Marmett, Júlia Mathias Reis, Bruna Silveira da Rosa, Carmem Lisiane Escouto de Souza, Tiago Chagas Dalcin, Sérgio Luís Amantéa, Frederico Viana Machado

**Affiliations:** 1 Ministério da Saúde, Programa de Desenvolvimento Institucional do Sistema Único de Saúde Porto Alegre, RS, Brazil; 2 Universidade Federal do Rio Grande do Sul, Programa de Pós-Graduação em Saúde Coletiva Porto Alegre, RS, Brazil

**Keywords:** Adolescent Pregnancy, Pregnancy, Mental Health, Depression, Anxiety, Embarazo Adolescente, Embarazo, Salud Mental, Depresión, Ansiedad

## Abstract

**Objective:**

To analyze the mental health of Brazilian adolescent mothers who use the Unified Health System (Sistema Único de Saúde, SUS).

**Methods:**

This is a multicenter study conducted with 583 adolescent mothers (10-19 years old). The participants responded to a questionnaire on sociodemographic variables, mental health and family support. The results were presented as absolute and relative frequencies. The association between outcome and variables was investigated using Poisson regression.

**Results:**

Some level of depression (61.9%) and anxiety (63.8%) was present in the adolescents. These symptoms were associated with a lack of family support (p-value<0.001), the loss of school friends (p-value<0.001), the diagnosis of postpartum depression (p-value<0.001), and the diagnosis of depression or anxiety before or during pregnancy (p-value<0.001). 15.9% of the adolescents diagnosed with mental health conditions were being treated. The main barriers identified were difficulties in access to psychological support, lack of interest and lack of encouragement.

**Conclusion:**

The study revealed worrying rates of depression and anxiety among teenage girls. The importance of family support was emphasized, the absence of which is linked to increased symptoms of mental distress. The impact of the loss of social ties and a history of mental health problems, such as postpartum depression, is highlighted. 15% of the adolescents had psychological monitoring, which makes evident the barriers to access to mental health. The results underline an urgent need for interventions and public policies to improve access to psychological support and to promote the mental health of this population.

## Resumen

**Objetivo:**

Analizar la salud mental de madres adolescentes brasileñas usuarias del Sistema Único de Salud.

**Métodos:**

Se trata de un estudio multicéntrico realizado con 583 madres adolescentes (10-19 años). Los participantes respondieron el cuestionario sobre variables sociodemográficas, salud mental y apoyo familiar. Los resultados se presentaron como frecuencias absolutas y relativas. La asociación entre el resultado y las variables se investigó mediante regresión de Poisson.

**Resultados:**

Se encontró algún nivel de depresión en el 61,9% de los adolescentes y ansiedad en el 63,8%. Estos síntomas se asociaron con falta de apoyo familiar (p-valor<0,001), pérdida de amigos de la escuela (p-valor<0,001), diagnóstico de depresión posparto (p-valor<0,001) y diagnóstico de depresión o ansiedad antes o durante el embarazo (p-valor<0,001). Los adolescentes diagnosticados con problemas de salud mental que estaban siendo monitoreados totalizaron el 15,9%. Las principales barreras identificadas fueron las dificultades para acceder al apoyo psicológico, el desinterés y la falta de estímulo.

**Conclusión:**

El estudio reveló tasas preocupantes de depresión y ansiedad entre los adolescentes. Se destacó la importancia del apoyo familiar, cuya ausencia se relaciona con un aumento de los síntomas de malestar mental. Se destacó el impacto negativo de la pérdida de vínculos sociales y los antecedentes de problemas de salud mental, como la depresión posparto. Los adolescentes que recibían apoyo psicológico representaban el 15,9%, lo que evidencia barreras para acceder a la salud mental. Los resultados destacaron la urgencia de intervenciones y políticas públicas para mejorar el acceso al apoyo psicológico y promover la salud mental de esta población.

**Ethical aspects**

This research respected ethical principles, having obtained the following approval data:

Research Ethics Committee: Hospital Moinhos de Vento

Opinion number: 6.179.211

Approval date: 12/7/2023

Certificate of Submission for Ethical Appraisal: 55465822.0.1001.5330

Informed Consent Form: Obtained from all participants prior to collection.

## Introduction

Teenage pregnancy is a significant phenomenon both in Brazil and worldwide, and it presents relevant challenges for public health and the well-being of adolescents (1). Annually, 1 million adolescents under 15 years old give birth, and 16 million adolescents between 15 and 19 years old also have babies (2). In low- and middle-income countries, 50.0% of these pregnancies are unplanned (3). The Brazilian pregnancy rate is 68.4 births for every 1,000 adolescents between 15 and 19 years old, which totals 364,734 births, of which 17,456 occur among girls aged 10 to 14 years (4). These statistics highlight the urgency of understanding and addressing this reality, since adolescents who experience motherhood have a higher prevalence of depression and anxiety (5). 

The mental health of adolescent mothers is a critical area of research because of its implications for the well-being of young women and the development of their children (6). It is estimated that 14.0% of adolescents in the world experience mental disorders (7). This makes clear the importance of understanding how teenage pregnancy can interact with these psychological conditions. In Brazil, teenage pregnancy is associated with psychological and social challenges, such as depression, conduct disorders and hyperactivity, which can negatively impact the development of mothers and their children (6,8). 

It is essential to recognize that teenage pregnancy is often stigmatized and associated with risky behaviors, which contributes to a simplistic view of the phenomenon and its underlying causes (1,9). This stigma can make it difficult to identify and appropriately address the mental health issues faced by teenage mothers. This underlines the need for a comprehensive approach that considers not only biological aspects, but also the social, cultural and historical contexts that influence this reality (10,11).

This article analyses the mental health of Brazilian adolescent mothers using the Unified Health System throughout their maternal trajectory, by means of comprehensive and multidisciplinary research, seeking to contribute to a better understanding of the challenges faced by adolescent girls and identifying effective strategies to promote their mental health and the well-being of their children. 

## Methods

### Study design

This is a multicenter study with cross-sectional design. 

### Context

The study was based on the research “Vulnerabilidades da Gestação Precoce no Brasil: impactos na mãe adolescente e na criança” (Vulnerabilities of Early Pregnancy in Brazil: Impacts on the Teenage Mother and her Child), carried out through the SUS Institutional Development Support Program. Data collection took place between August 2022 and May 2023, by means of interviews in the adolescents home or during hospital appointments. The study followed the guidelines and rules of the Resolution 466/12 of the National Health Council (12). 

### Participants

Mothers aged between 10 and 19 years old who gave birth in the selected institutions between June 2021 and June 2022 were included. All signed the Informed Consent Form or the Assent Form, and all were fluent in Portuguese, being able to understand the objectives of the study. Adolescents who met the inclusion criteria were invited to participate by telephone contact. The choice to include mothers who gave birth between June 2021 and June 2022 aimed to avoid the period of hospital restrictions due to the COVID-19 pandemic, which ensured adequate monitoring and good physical and mental conditions for the study. 

### Variables

For the diagnostic outcome of depression and anxiety, the dichotomous variable from the Depression, Anxiety and Stress Questionnaire (DASS-21) was created; adolescent mothers with responses other than “normal” in the depression and anxiety domains were classified as “yes”. The models included demographic variables, education, employment, family support, and general, gestational, and mental health as independent variables. The following variables were used as independent covariates.

- Demographics: mother’s age (years); marital status (single, married, common-law marriage); race/skin color (white, non-white); education (elementary school, high school, higher education); employment status (working, unemployed, never worked); age of the child’s father (years).
- Health of the adolescent mother before pregnancy (diagnosed diseases): depression; anxiety; psychiatric disorders; infectious diseases; hematological; nephropathies; and malnutrition (yes, no).
- Postpartum depression: Did the teenage mother have postpartum depression? (yes, no).
- Interruption of studies: stopped studying during pregnancy; and was studying when the son was born (yes, no).
- Support from the child’s father and family: receives emotional support, support in daily life, financial support from the child’s father and family support (yes, no).
- Family support: receives financial support, support in daily life, health support and emotional support (yes, no)
- Involvement of the child’s father in the pregnancy: How was paternal involvement during pregnancy? (good, excellent, regular, bad).
- Mental Health Diagnoses: Has any doctor or mental health professional given the adolescent mother a diagnosis of depression or anxiety? (no, before the 2021/2022 pregnancy, during the 2021/2022 pregnancy).
- Factors that influenced pregnancy: got pregnant by accident; wanted to be a mother; wanted to have another child; wanted to build a family; wanted to leave her parents/guardians’ house; wanted to get married; got pregnant because she thought she would be more respected after becoming a mother; didn’t know how to avoid having children; couldn’t afford contraceptives; got married early; had no other option; it was a life project; her husband/partner wanted to have children soon; wanted to develop greater maturity; her partner didn’t want to use a condom; didn’t know where to get contraceptives; was a victim of abuse; the contraceptive failed (true, false).
- Changes that occurred after pregnancy: your life became more difficult; your life became more organized; you became more respected; your relationship with your husband/partner improved; your husband/partner abandoned you; you were rejected by your family; dropped out of school/technical course/college; wanted to study to give the baby a good future; your life improved because now you have your own home; now you have a reason to live; this was the worst period of your life; you started to feel better about yourself; she got married; it became difficult to get a job and staying at work; lost friends/schoolmates; created new friendships or got closer to women who are also mothers; It’s gotten harder to date (true, false).

### Data source

Five reference hospitals were selected, located in capitals of each of the five regions of Brazil, which presented high indicators of live births to teenage mothers (4). The selection of hospitals was based on their link with the SUS and the certification by the Baby-Friendly Hospital Initiative.

### Bias

The information was obtained through a structured form prepared by the study coordinating team. Trained and certified female interviewers conducted the interviews to minimize the possibility of bias. The interviews were monitored and the database was checked.

### Study size

The sample required for the study was 583 participants, taking into account that 26.3% of adolescents could present anxiety and depression, in addition to the possibility that 10.0% of the participants would not complete the questionnaire. To ensure that the study was able to detect real differences, the sample size used ensures high probability of identifying these differences, with 80% power and a 95% confidence level (13). The sampling was non-probabilistic, including participants proportionally to the number of live births to adolescent mothers by region of Brazil.

### Statistical methods

Data analysis began with the creation of the database in Excel, and the information was encoded and validated by double entry. Categorical variables were summarized in absolute and relative frequencies, and continuous variables in mean and standard deviations or medians and interquartile range. The Shapiro-Wilk test assessed normality. Some variables were recategorized to facilitate interpretation, such as the variable race/skin color (white and non-white) and education (elementary, secondary, higher education and do not know), excluding responses “do not want to answer” or “do not remember”.

The validation of the DASS-21 scale into Brazilian Portuguese was carried out by translation-back-translation with 242 participants, showing high adequacy to the model (Kaiser-Meyer-Olkin [KMO] test of 0.949) and good internal consistency (Cronbach’s alpha: 0.92 for depression, 0.90 for stress and 0.86 for anxiety). Correlations with the Beck scales and the Lipp Stress Symptoms Inventory, and the factor analysis, confirmed the validity of the DASS-21 to separately assess depression, anxiety and stress (14).

The DASS-21 Scale is composed of three subscales, each with seven statements, assessing emotional states on the Likert scale from 0 to 3. Intensity levels (normal, mild, moderate, severe, and extremely severe) indicate the presence of symptoms (13). Factor analysis confirmed the structure of three distinct factors (14). “Normal” means no symptoms, and results were presented as presence or absence of symptoms and in levels of intensity. The total score is the sum of the items in each subscale.

The association between the variables and the prevalence of anxiety and depression was analyzed by Poisson regression models with robust variance, estimating prevalence ratios and correcting overdispersion problems, which ensured precise and reliable 95%CI (15).

## Results

583 adolescent mothers from all regions of Brazil participated in the research. Socioeconomic, educational, occupational characteristics, diagnosis of depression and anxiety and access to mental health treatment were analyzed (Table 1). The average age was 18 years (16-19), 83.7% declared themselves non-white. The majority were single (85.8%) and 69.4% participated in social programs, with an average income of R$ 1,212.00 (R$ 880-1,900). The average age of the child’s fathers was 22 years (20-25). The percentage of mothers who were studying was 20.5%, with complete or incomplete secondary education being the most common (63.7%). Among those who didn’t study, 44.7% mentioned motherhood as the reason and 51.4% interrupted their studies due to pregnancy. The majority were unemployed (48.7%), with 15.9% working in some capacity.

**Table 1:** Absolute and relative frequencies (%) of socioeconomic, educational, occupational and mental health characteristics of adolescent mothers. Brazil, 2022-2023 (n=583)

Variable	n (%)
**Race/skin color**	
White	95 (16.3)
Non-white	488 (83.7)
**Marital status**	
Single	500 (85.8)
Married	66 (11.3)
Common-law marriage	17 (2.9)
**Is a beneficiary in government social programs**	403 (69.4)
**Currently studying**	120 (20.5)
**Level of education**	
Elementary education (complete/incomplete)	199 (34.1)
High school (complete/incomplete)	371 (63.7)
Higher education (complete/incomplete)	13 (2.2)
**Reasons for not studying**	
Gestation/be a mothers	261 (44.7)
Stopped studying during pregnancy	300 (51.4)
**Occupational status**	
Working	93 (15.9)
Unemployed	284 (48.7)
Never worked	206 (35.3)
**Diagnosis of depression and** anxiety	
Before pregnancy	88 (15.0)
During pregnancy	44 (7.5)
Attends mental health consultations	21 (15.9)
**Reasons for not undergoing mental health monitoring**	
Unable to access psychological support in the Unified Health System	26 (29.5)
No interest	23 (26.1)
Was not encouraged to continue monitoring	22 (25.0)
**DASS-21^a^ condition**	
Stress	325 (55.7)
Anxiety	372 (63.8)
Depression	361 (61.9)

^a^Depression, anxiety and stress scale.

132 adolescents had a diagnosis of depression or anxiety (22.6%); 88 were diagnosed before the pregnancy, and 44 had received the diagnosis during the pregnancy. Among those diagnosed, 21 (15.9%) were undergoing psychological monitoring. Those who received the diagnosis before pregnancy mentioned difficulties in accessing the psychologist through the SUS (26 [29.5%]), disinterest (23 [26.1%]) or lack of encouragement (22 [25.0%]) (Table 1). The prevalence of symptoms of depression, anxiety and stress was 61.9%, 63.8% and 55.7%.

The prevalence of depression and anxiety was 25.0% higher among adolescents in common-law marriage. Those who had not previously been diagnosed with depression presented 15.0% fewer symptoms. There was a significant association between mental health and the diagnosis of postpartum depression (p-value<0.001). Adolescents without postpartum depression presented 24.0% less prevalence of depression or anxiety.

The teenagers responded to statements with “true” or “false” (Supplementary Table 1). Those who said that life became more difficult after pregnancy presented 22.0% higher prevalence of depression/anxiety (p-value 0.002). Those who lost friends/schoolmates had 23.0% higher prevalence of positive results in DASS-21 (p-value 0.031). The adolescents who felt more respected presented 10.0% lower prevalence of depression/anxiety (p-value<0.001). 

The analysis of the variables investigated showed that the lack of family support increased the prevalence of symptoms (p-value<0.001). Adolescents without family support presented 32.0% higher prevalence of depression/anxiety. Those who felt a lack of support in their daily life with the baby presented 23.0% higher prevalence (Table 3). Adolescents who considered the baby father’s involvement during pregnancy to be excellent presented 15.0% lower prevalence of depression/anxiety (p-value 0.014). Those diagnosed with depression before or during pregnancy presented 36.0% more prevalence of symptoms at the time of the interview (Table 3).

**Table 2:** Prevalence ratio (PR) and 95% confidence interval (95%CI) related to anxiety and depression in adolescent mothers. Brazil, 2022-2023 (n=583)

Variable	PR (95%CI)^c^	p-value	PR (95%CI)^c^	p-value
Model 1^a^ Demographics^b^	Crude model^b^		Adjusted model^a^	
**Mother’s age** (years)	0.96 (0.92; -1.00)	0.031	0.97 (0.94; 1.00)	0.094
**Marital status^c^**				
Single	1.00	-	1.00	-
Married	0.96 (0.81; 1.14)	0.621	0.95 (0.80; 1.13)	0.565
Common-law marriage	1.25 (1.04; 1.51)	0.015	1.23 (1.03; 1.46)	0.024
**Race/skin color**				
White	1.00	-	-	-
Non-white	0.92 (0.82; 1.04)	0.206	-	-
Education				
Elementary (complete/incomplete)	1.00	-	-	-
High School (complete/incomplete)	1.05 (0.93; 1.18)	0.435	-	-
Higher education (complete/incomplete)	0.79 (0.47; 1.32)	0.367	-	-
**Occupational status**				
Working	1.00	-	-	-
Unemployed	1.01 (0.87; 1.17)	0.875	-	-
Never worked	0.91 (0.78; 1.07)	0.252	-	-
**Age of the child’s father**	1.00 (0.99; 1.01)	0.665	-	-
**Education level of the child’s father**				
Incomplete elementary school	1.00	-	-	-
High School (complete/incomplete)	0.93 (0.83; 1.05)	0.229	-	
Higher education (complete/incomplete)	0.97 (0.73; 130)	0.852		
Does not know	0.90 (0.76; 1.07)	0.227	-	-
Model 2^a^ – Health of the adolescent mother before pregnancy	Crude model		Adjusted model	
Depression				
Yes	1.00	-	1.00	-
No	0.84 (0.78; 0.92)	<0.001	0.85 (0.78; 0.92)	<0.001
**Anxiety**				
Yes	1.00	-	1.00	-
No	0.75 (0.68; 0.82)	<0.001	0.75 (0.68; 0.82)	<0.001
**Psychiatric disorders**				
Yes	1.00	-	-	-
No	1.04 (0.99; 1.10)	0.103	-	-
Infectious				
Yes	1.00	-	-	-
No	0.93 (0.69; 1.26)	0.653	-	-
Hematological				
Yes	1.00	-	-	-
No	0.89 (0.69; 1.15)	0.382	-	-
Nephropathies				
Yes	1.00	-	-	-
No	1.51 (0.63; 3.63)	0.356	-	-
Malnutrition				
Yes	1.00	-	-	-
No	1.02 (0.76; 1.37)	0.897	-	-
Model 3^a^ – Postpartum mental health diagnosis^b^ – d	Crude model^b^		Adjusted model^a^	
**Postpartum depression**				
Yes	1.00	-	-	-
No	0.76 (0.69; 0.83)	<0.001	-	-

^a^The models presented in this table have as a variable the response “Diagnosis of depression/anxiety (DASS-21); ^b^Models 1, 2 and 3 include different independent variables related to demographic information, diagnoses before pregnancy and postpartum mental health diagnosis;^c^The effect of each explanatory variable was assessed through PR with 95%CI.

**Table 3:** Prevalence rate (PR) and 95% confidence interval (95%CI) related to anxiety, depression and stress before and during pregnancy in adolescent mothers. Brazil, 2022-2023 (n=583)

Variable	PR (95%CI)^c^	p-value	PR (95%CI)^c^	p-value
Model 4^a^ – Interruption of studies	Crude modelB		Adjusted model^a^	
**Stopped studying during pregnancy**				
No	1.00	-	-	-
Yes	1.09 (0.98; 1.21)	0.096	-	-
**Was studying when child was born**				
No	1.00	-	-	-
Yes	0.96 (0.86; 1.07)	0.431	-	-
Model 5^a^ – Support from own family and the child’s father^b^	Crude model		Adjusted model	
**Gets emotional support**				
Yes	1.00	-	-	-
No	1.13 (0.96; 1.32)	0.135	-	-
**Gets support in daily life**				
Yes	1.00	-	-	-
No	1.09 (0.94; 1.26)	0.245	-	-
**Receives financial support**				
Yes	1.00	-	-	-
No	0.89 (0.77; 1.03)	0.114	-	-
**Receives family support**				
Yes	1.00	-	-	-
No	1.32 (1.19; 1.46)	<0.001	-	-
Model 6^a^ – Family support	Crude model		Adjusted model	
**Receives financial support**				
Yes	1.00	-	-	-
No	1.04 (0.92; 1.18)	0.535	-	-
**Gets support in daily life**				
Yes	1.00	-	1.00	-
No	1.21 (1.07; 1.37)	0.003	1.23 (1.11; 1.37)	<0.001
**Get health support**				
Yes	1.00	-	-	-
No	1.01 (0.89; 1.15)	0.911	-	-
**Gets emotional support**				
Yes	1.00	-	1.00	-
No	1.13 (1.00; 1.29)	0.059	1.14 (1.00; 1.30)	0.055
Model 7^a^ – Mother’s involvement in the gestation^b^	Crude model^b^		Adjusted model^a^	
**Involvement of the child’s father in the pregnancy**				
Good	1.00	-	-	-
Excellent	0.85 (0.74; 0.97)	0.014	-	-
Regular	1.08 (0.94; 1.24)	0.296	-	-
Bad	0.97 (0.82; 1.14)	0.690	-	-
Model 8^a^ – Mental health diagnosis^b^	Crude model		Adjusted model	
**Depression/Anxiety diagnosis**				
No	1.00	-	-	-
Before the 2021/2022 pregnancy	1.36 (1.24; 1.49)	<0.001	-	-
During the 2021/2022 gestation	1.36 (1.22; 1.53)	<0.001	-	-

^a^The models presented in this table have as variable a response of “Diagnosis of depression/anxiety (DASS-21)”; ^b^The models 4, 5, 6, 7, and 8 include different independent variables related to information on interruption of studies, to the support from the child’s father, to the family support, paternal involvement during pregnancy and, to the mental health diagnosis; ^c^The effect of each explanatory variable was assessed through PR with 95%CI.

## Discussion

The findings of this research reveal an alarming reality regarding mental health among adolescent mothers in Brazil and evidence the relationship between socioeconomic conditions and symptoms of depression and anxiety. Most participants were 18 years old, single and lived in a vulnerable socioeconomic situation, with high participation in social programs. Low levels of education and interruption of studies, often due to motherhood, worsen mental health. The prevalence of symptoms was higher among those without family support and with difficulties accessing mental health services. Paternal support during pregnancy was a protective factor, indicating that positive family relationships can improve emotional health. These results reinforce the need for interventions that address both the psychological support and the social conditions of this population. 

This study has limitations. The lack of stratification of adolescents into two age groups (10-14 years and 15-19 years) limits the analysis, especially due to the inadequacy of the sample of mothers under 14 years of age. Although the sample included adolescents from different regions, all participants served had the Brazilian Unified Health System (SUS) as the paying entity, which keeps homogeneity. The lack of regionalized assessment limits understanding variations in the context of Brazilian regions. The interviews were self-reported, which may introduce recall bias. Most pregnancies occurred during the health emergency of COVID-19, which may have impacted access to health services and caused social isolation. 

The data reveals that 361 adolescents (61.9%) presented some level of depression, 372 (63.8%) manifested anxiety and 325 (55.7%) exhibited stress. These results were worrying and suggest significantly higher prevalence of mental health problems among adolescent mothers when compared with an analysis conducted with Brazilian students, which found 11.7% cases of depression, 10.2% of anxiety and 11.3% of stress, using the same metric as DASS-21 (16). Although the analysis mentioned was not carried out with pregnant adolescents or those with recent births, it shows differences in the impact of pregnancy on mental health, which reflected the additional pressures that motherhood imposes. 

The anxiety, depression and stress levels were associated to the diagnosis of postpartum depression (p-value<0.001), increased difficulties after childbirth (p-value 0.002) and loss of school friends (p-value<0.001). These associations indicate that postpartum difficulties could be linked to the increased responsibilities of motherhood, while the loss of friends reflects the incompatibility between the previous routine and the new demands (17,18). The social support network was crucial in this period, and its absence could intensify isolation and depression (19). 

In the Brazilian Northeast, 56,5% of the adolescents showed a low risk for depression, but this research had a smaller sample and the participants were pregnant, which can explain the differences found (20). Internationally, up to 29.0% of adolescent mothers have depression, which reinforces the need for specialized attention (21). Comparing the mental health of pregnant and non-pregnant adolescents, 24.6% of pregnant women showed signs of mental problems, while 27.3% of non-pregnant women had mental issues (5). Differences in assessment instruments contribute to the contrasting results.

It was observed that teenagers who felt more respected after pregnancy presented prevalence 10.0% lower of positive levels of anxiety and depression (p-value 0.031), which demonstrates that adolescence pregnancy can also contribute in a protective manner for maternal mental health. This data relates to the perception of self-efficacy and self-esteem, in which the view of value and respect proves to be protective against depression in the first months after pregnancy (19). 

Among the 583 adolescents participating in the survey, 132 (22.6%) were diagnosed with anxiety or depression at some point in their lives. This data is higher than that found in Pernambuco, where 15.6% of participants were diagnosed with a mental disorder before pregnancy (22). This study demonstrated that previous diagnosis (p-value<0.001) or a diagnosis during pregnancy (p-value<0.001) has an important association with levels of depression, anxiety and stress at the time of the interview. A history of mental disorders before pregnancy was a significant risk factor for the development of new symptoms during pregnancy, affecting the mental health of women of different age groups (23). For adolescents, this concern becomes even more alarming, given the emotional vulnerability characteristic of this phase of development (19). 

Despite the significant proportion of adolescents presenting signs of anxiety or depression at the time of the interview, only 21 (15.9%) reported attending mental health consultations. Those who had been diagnosed for a longer time (before pregnancy) mentioned difficulties in accessing the psychologist through the SUS (26 [29.5%]), disinterest (23 [26.1%]) or lack of encouragement to maintain follow-up (22 [25.0%]). A significant part of mothers with anxiety (48.0%) and depression (70.0%) continued facing psychological suffering due to not receiving adequate guidance and support (24). Many did not seek help due to social stigmas or difficulty in accessing resources, resulting in low adherence to treatment and monitoring of their condition (25,26).

The normalization of psychological suffering in adolescent mothers is a challenge that needs to be addressed with public health strategies that prioritize awareness and education in mental health. The scarcity of financial resources contributes to limited access to mental health (27,28). This scenario is alarming, since mothers who experience psychological distress before the birth of their baby have a high probability of continuing to present or may have worse symptoms after childbirth (24,28). Professional attention and appropriate referral to specialized care are essential for improving maternal mental health (29).

In the Brazilian scenario, girls who do not have support from their partner during pregnancy were 1.4 times more likely to develop mental disorders (30). Additionally, 57.0% of adolescent mothers without social and family support during or after pregnancy have depressive symptoms four years after giving birth (31). The data from this study corroborates the perception that social support is essential so that adolescents do not feel overwhelmed by new responsibilities, functioning as a form of mental health protection (24,31). The involvement of the child’s father in care can contribute to outcomes related to the mental health of adolescents and protect the baby from socioemotional risks involved in cases of maternal depression (21).

This study highlights the worrying rates of depression, anxiety and stress among adolescent mothers treated at the SUS. Associations with social support, family planning and clinical diagnosis offer important analyses for effective interventions. It is necessary to plan health policies that prevent harm and promote mental health, including emotional support and adequate treatment. The lack of access to mental health in the SUS is an urgent concern that requires reassessment. Actions should be implemented in an integrated manner, involving health services, community and family, to ensure that adolescent mothers receive the support they need during this critical period in their lives.

## Data Availability

The database and analysis codes used in the research are available at: https://drive.google.com/drive/folders/1ER03g1jnm9pAyZYmwBmU5gxzHBaf7fBu?usp=drive_link.

## Supplementary material

Supplementary Table 1 and Supplementary Table 2.
