# Supplementary material for: A multicenter study on the mental health of Brazilian adolescent mothers, 2024
Source: Epidemiol Serv Saude. 2025 Sep 8;34:e20240226. doi: 10.1590/S2237-96222025v34e20240226.en (PMC12435885; doi:10.1590/S2237-96222025v34e20240226.en)
Supplement: Supplementary file 1 [file 2237-9622-ress-34-e20240226-suppl01-en.pdf]

Supplementary Table 1. Razão prevalence (PR) and interval crude and adjusted 95% confidence intervals (95%CI) related to factors that influenced pregnancy and changes in the lives of teenage mothers. Brazil, 2022-2023 (n=583)

**Variable**	**PR (95%CI)[a]**	**p-value**	**PR (95%CI)[a]**	**p-value**
	**Crude model**		**Adjusted model**	
**Factors that influenced pregnancy**				
**Got pregnant by accident**				
False	1.00	-	-	-
True	0.96 (0.85;1.08)	0.449	-	-
**Wanted to be a mother**				
False	1.00	-	-	-
True	0.91 (0.77;1.07)	0.261	-	-
**Wanted to have another child**				
False	1.00	-	-	-
True	0.94 (0.76;1.16)	0.55	-	-
**Wanted to have a family**				
False	1.00	-	-	-
True	1.03 (0.88;1.21)	0.704	-	-
**Wanted to leave parents/guardians' house**				
False	1.00	-	-	-
True	1.02 (0.86;1.22)	0.795	-	-
**Wanted to get married**				
False	1.00	-	-	-
True	0.99 (0.83;1.19)	0.944	-	-
**Got pregnant because she thought she would be more respected after becoming a mother**				

Variable	PR (95%CI)[a]	p-value	PR (95%CI)[a]	p-value
	Crude model		Adjusted model	
False	1.00	-	-	-
True	1.08 (0.92;1.26)	0.341	-	-
**Didn't know how to avoid children**				
False	1.00	-	-	-
True	0.98 (0.85;1.12)	0.754	-	-
**Could not afford to buy contraceptives**				
False	1.00	-	-	-
True	1.02 (0.86;1.20)	0.846	-	-
**Married early**				
False	1.00	-	-	-
True	0.99 (0.87;1.13)	0.915	-	-
**Had no other option**				
False	1.00	-	-	-
True	1.06 (0.90;1.25)	0.489	-	-
**It was a life project**				
False	1.00	-	-	-
True	1.02 (0.88;1.18)	0.763	-	-
**The husband/partner wanted to have children soon**				
False	1.00	-	-	-
True	1.08 (0.94;1.24)	0.254	-	-
**Wanted to develop greater maturity**				
False	1.00	-	-	-
True	1.10 (0.95;1.26)	0.193	-	-
**Partner did not want to use a condom**				
False	1.00	-	-	-

Variable	PR (95%CI)[a]	p-value	PR (95%CI)[a]	p-value
	**Crude model**		**Adjusted model**	
True	1.05 (0.94;1.18)	0.392	-	-
**Did not know where to get a contraceptive**				
False	1.00	-	-	-
True	0.97 (0.79;1.18)	0.734	-	-
**Was a victim of abuse**				
False	1.00	-	-	-
True	0.82 (0.29;2.31)	0.704	-	-
**The contraceptive failed**				
False	1.00	-	-	-
True	1.04 (0.94;1.16)	0.415	-	-
**Changes that occurred after pregnancy**				
**Life has become more difficult**				
False	1.00	-	1.00	-
True	1.20 (1.05;1.37)	0.007	1.22 (1.08;1.38)	0.002
**Life has become more organized**				
False	1.00	-	-	-
True	1.03 (0.92;1.15)	0.564	-	-
**Became more respected**				
False	1.00	-	1.00	-
True	0.89 (0.80;0.99)	0.039	0.90 (0.82;0.99)	0.031
**Relationship with husband/partner has improved**				
False	1.00	-	-	-
True	0.91 (0.81;1.03)	0.129	-	-
**Husband/partner has abandoned her**				
False	1.00	-	-	-

Variable	PR (95%CI)[a]	p-value	PR (95%CI)[a]	p-value
	**Crude model**		**Adjusted model**	
True	0.93 (0.82;1.05)	0.252	-	-
**Was rejected by her family**				
False	1.00	-	-	-
True	1.02 (0.88;1.19)	0.759	-	-
Developed a better relationship with own family				
False	1.00	-	-	-
True	0.92 (0.82;1.04)	0.179	-	-
**Dropped out of school/technical course/college**				
False	1.00	-	-	-
True	0.95 (0.86;1.05)	0.315	-	-
**Wanted to study to give her baby a good future.**				
False	1.00	-	-	-
True	1.33 (0.93;1.88)	0.115	-	-
**Life has improved because she have created her own home**				
False	1.00	-	-	-
True	1.02 (0.90;1.15)	0.727	-	-
**She now has a reason to live**				
False	1.00	-	-	-
True	1.11 (0.84;1.48)	0.462	-	-
**It was the worst period of her life**				
False	1.00	-	-	-
True	1.04 (0.92;1.17)	0.526	-	-
**She started to feel better about herself**				
False	1.00	-	-	-
True	1.05 (0.92;1.20)	0.434	-	-

Variable	PR (95%CI)[a]	p-value	PR (95%CI)[a]	p-value
	Crude model		Adjusted model	
**Got married**				
False	1.00	-	-	-
True	0.94 (0.83;1.07)	0.355	-	-
**Getting a job and remaining employed got harder**				
False	1.00	-	-	-
True	1.03 (0.93;1.15)	0.589	-	-
**Lost group of friends/schoolmates**				
False	1.00	-	1.00	-
True	1.21 (1.07;1.36)	0.002	1.23 (110;1.38)	<0.001
**Made new friends or got closer to women who are also mothers**				
False	1.00	-	-	-
True	0.91 (0.81;1.03)	0.146	-	-
**It's gotten harder to date**				
False	1.00	-	-	-
True	1.08 (0.98;1.20)	0.116	-	-

Note:[a]The effect of each explanatory variable was assessed through PR with 95%CI.
