# Supplementary material for: A multicenter study on the mental health of Brazilian adolescent mothers, 2024
Source: Epidemiol Serv Saude. 2025 Sep 8;34:e20240226. doi: 10.1590/S2237-96222025v34e20240226.en (PMC12435885; doi:10.1590/S2237-96222025v34e20240226.en)
Supplement: Supplementary file 2 [file 2237-9622-ress-34-e20240226-suppl01-pt.pdf]

Tabela Suplementar 1. Razão de prevalência (RP) e intervalo de confiança de 95% (IC95%) brutos e ajustados relacionados a fatores que influenciaram a gestação e mudanças na vida das mães adolescentes. Brasil, 2022-2023 (n=583)

**Variável**	**RP (IC95%)[a]**	**p-valor**	**RP (IC95%)[a]**	**p-valor**
	**Modelo bruto**		**Modelo ajustado**	
**Fatores que influenciaram a gestação**				
**Engravidou sem querer**				
Falso	1,00	-	-	-
Verdadeiro	0,96 (0,85; 1,08)	0,449	-	-
**Queria ser mãe**				
Falso	1,00	-	-	-
Verdadeiro	0,91 (0,77; 1,07)	0,261	-	-
**Queria ter outro filho**				
Falso	1,00	-	-	-
Verdadeiro	0,94 (0,76; 1,16)	0,55	-	-
**Queria construir uma família**				
Falso	1,00	-	-	-
Verdadeiro	1,03 (0,88; 1,21)	0,704	-	-
**Queria sair da casa dos pais/responsáveis**				
Falso	1,00	-	-	-
Verdadeiro	1,02 (0,86; 1,22)	0,795	-	-
**Queria casar**				
Falso	1,00	-	-	-
Verdadeiro	0,99 (0,83; 1,19)	0,944	-	-
**Engravidou porque achava que seria mais respeitada após ser mãe**				
Falso	1,00	-	-	-

Variável	RP (IC95%)[a]	p-valor	RP (IC95%)[a]	p-valor
	Modelo bruto		Modelo ajustado	
Verdadeiro	1,08 (0,92; 1,26)	0,341	-	-
**Não sabia como evitar filhos**				
Falso	1,00	-	-	-
Verdadeiro	0,98 (0,85; 1,12)	0,754	-	-
**Não tinha condições de comprar contraceptivos**				
Falso	1,00	-	-	-
Verdadeiro	1,02 (0,86; 1,20)	0,846	-	-
**Casou cedo**				
Falso	1,00	-	-	-
Verdadeiro	0,99 (0,87; 1,13)	0,915	-	-
**Não tinha outra opção**				
Falso	1,00	-	-	-
Verdadeiro	1,06 (0,90; 1,25)	0,489	-	-
**Era um projeto de vida**				
Falso	1,00	-	-	-
Verdadeiro	1,02 (0,88; 1,18)	0,763	-	-
**O marido/companheiro queria ter filhos logo**				
Falso	1,00	-	-	-
Verdadeiro	1,08 (0,94; 1,24)	0,254	-	-
**Queria desenvolver maior maturidade**				
Falso	1,00	-	-	-
Verdadeiro	1,10 (0,95; 1,26)	0,193	-	-
**O parceiro não queria usar camisinha**				
Falso	1,00	-	-	-
Verdadeiro	1,05 (0,94; 1,18)	0,392	-	-

Variável	RP (IC95%)[a]	p-valor	RP (IC95%)[a]	p-valor
	**Modelo bruto**		**Modelo ajustado**	
**Não sabia onde conseguir um anticoncepcional**				
Falso	1,00	-	-	-
Verdadeiro	0,97 (0,79; 1,18)	0,734	-	-
**Foi vítima de abuso**				
Falso	1,00	-	-	-
Verdadeiro	0,82 (0,29; 2,31)	0,704	-	-
**O contraceptivo falhou**				
Falso	1,00	-	-	-
Verdadeiro	1,04 (0,94; 1,16)	0,415	-	-
**Mudanças que ocorreram após a gestação**				
**Sua vida ficou mais difícil**				
Falso	1,00	-	1,00	-
Verdadeiro	1,20 (1,05; 1,37)	0,007	1,22 (1,08; 1,38)	0,002
**Sua vida ficou mais organizada**				
Falso	1,00	-	-	-
Verdadeiro	1,03 (0,92; 1,15)	0,564	-	-
**Passou a ser mais respeitada**				
Falso	1,00	-	1,00	-
Verdadeiro	0,89 (0,80; 0,99)	0,039	0,90 (0,82; 0,99)	0,031
**Sua relação com o marido/companheiro melhorou**				
Falso	1,00	-	-	-
Verdadeiro	0,91 (0,81; 1,03)	0,129	-	-
**Seu marido/companheiro lhe abandonou**				
Falso	1,00	-	-	-
Verdadeiro	0,93 (0,82; 1,05)	0,252	-	-

Variável	RP (IC95%)[a]	p-valor	RP (IC95%)[a]	p-valor
	Modelo bruto		Modelo ajustado	
**Foi rejeitada pela família**				
Falso	1,00	-	-	-
Verdadeiro	1,02 (0,88; 1,19)	0,759	-	-
Desenvolveu uma relação melhor com a sua família				
Falso	1,00	-	-	-
Verdadeiro	0,92 (0,82; 1,04)	0,179	-	-
**Abandonou a escola/curso técnico/faculdade**				
Falso	1,00	-	-	-
Verdadeiro	0,95 (0,86; 1,05)	0,315	-	-
**Teve vontade de estudar para dar um bom futuro para o bebê**				
Falso	1,00	-	-	-
Verdadeiro	1,33 (0,93; 1,88)	0,115	-	-
**Sua vida melhorou porque formou o seu próprio lar**				
Falso	1,00	-	-	-
Verdadeiro	1,02 (0,90; 1,15)	0,727	-	-
**Passou a ter um motivo para viver**				
Falso	1,00	-	-	-
Verdadeiro	1,11 (0,84; 1,48)	0,462	-	-
**Foi o pior período da sua vida**				
Falso	1,00	-	-	-
Verdadeiro	1,04 (0,92; 1,17)	0,526	-	-
**Passou a ficar melhor consigo mesma**				
Falso	1,00	-	-	-
Verdadeiro	1,05 (0,92; 1,20)	0,434	-	-
**Casou**				

**Variável**	**RP (IC95%)[a]**	**p-valor**	**RP (IC95%)[a]**	**p-valor**
	**Modelo bruto**		**Modelo ajustado**	
Falso	1,00	-	-	-
Verdadeiro	0,94 (0,83; 1,07)	0,355	-	-
**Dificultou a obtenção e permanência no trabalho**				
Falso	1,00	-	-	-
Verdadeiro	1,03 (0,93; 1,15)	0,589	-	-
**Perdeu a turma de amigos/escola**				
Falso	1,00	-	1,00	-
Verdadeiro	1,21 (1,07; 1,36)	0,002	1,23 (1,10; 1,38)	<0,001
**Criou novas amizades ou se aproximou de mulheres que também são mães**				
Falso	1,00	-	-	-
Verdadeiro	0,91 (0,81; 1,03)	0,146	-	-
**Ficou mais difícil namorar**				
Falso	1,00	-	-	-
Verdadeiro	1,08 (0,98; 1,20)	0,116	-	-

Nota: [a]O efeito de cada variável explicativa foi avaliado por meio de RP com IC95%.
