# Supplementary material for: A multicenter study on the mental health of Brazilian adolescent mothers, 2024
Source: Epidemiol Serv Saude. 2025 Sep 8;34:e20240226. doi: 10.1590/S2237-96222025v34e20240226.en (PMC12435885; doi:10.1590/S2237-96222025v34e20240226.en)
Supplement: Translation

# Translation: Estudo multicêntrico sobre a saúde mental de mães adolescentes brasileiras, 2024

**DOI:** 10.1590/S2237-96222025v34e20240226.pt

**Authors:** Amanda Ferreira de Carvalho, Daniela Dal Forno Kinalski Guaranha, Bruna Marmett, Júlia Mathias Reis, Bruna Silveira da Rosa, Carmem Lisiane Escouto de Souza, Tiago Chagas Dalcin, Sérgio Luís Amantéa, Frederico Viana Machado

**Affiliations:** 1 Ministério da Saúde, Programa de Desenvolvimento Institucional do Sistema Único de Saúde Porto Alegre, RS, Brasil; 2 Universidade Federal do Rio Grande do Sul, Programa de Pós-Graduação em Saúde Coletiva Porto Alegre, RS, Brasil

**Keywords:** Gravidez na Adolescência, Gravidez, Saúde Mental, Depressão, Ansiedade

## Abstract

**Objetivo:**

Analisar a saúde mental das mães adolescentes brasileiras usuárias do Sistema Único de Saúde.

**Métodos:**

Trata-se de um estudo multicêntrico realizado com 583 mães adolescentes (10-19 anos). As participantes responderam ao questionário sobre variáveis sociodemográficas, saúde mental e apoio familiar. Os resultados foram apresentados como frequências absolutas e relativas. A associação entre desfecho e variáveis foram investigadas por meio de regressão de Poisson.

**Resultados:**

Houve algum nível de depressão em 61,9% das adolescentes e de ansiedade em 63,8%. Esses sintomas associaram-se à falta de apoio familiar (p-valor<0,001), à perda de amigos da escola (p-valor<0,001), ao diagnóstico de depressão pós-parto (p-valor<0,001) e ao diagnóstico de depressão ou ansiedade antes ou durante a gestação (p-valor<0,001). As adolescentes diagnosticadas com problemas de saúde mental que estavam em acompanhamento totalizaram 15,9%. As principais barreiras identificadas foram dificuldades de acesso ao suporte psicólogo, desinteresse e falta de estímulo.

**Conclusão:**

O estudo revelou preocupantes taxas de depressão e ansiedade entre as adolescentes. Enfatizou-se a importância do apoio familiar, cuja ausência está ligada ao aumento dos sintomas de sofrimento mental. Destacou-se o impacto negativo da perda de vínculos sociais e do histórico de problemas de saúde mental, como a depressão pós-parto. As adolescentes que estavam em acompanhamento psicológico totalizaram 15,9%, o que evidenciou barreiras de acesso à saúde mental. Os resultados sublinharam a urgência de intervenções e políticas públicas para melhorar o acesso ao suporte psicológico e promover a saúde mental dessa população.

**Aspectos éticos**

A presente pesquisa respeitou os princípios éticos, obtendo os seguintes dados de aprovação:

Comitê de ética em pesquisa: Hospital Moinhos de Vento

Número do parecer: 6.179.211

Data de aprovação: 12/7/2023

Certificado de apresentação de apreciação ética: 55465822.0.1001.5330

Registro do consentimento livre e esclarecido: Obtido de todos os participantes antes da coleta.

## Introdução

A gravidez na adolescência é um fenômeno significativo tanto no Brasil quanto no mundo e apresenta desafios relevantes para a saúde pública e o bem-estar das adolescentes (1). Anualmente, no mundo, ocorrem 1 milhão de partos de adolescentes com menos de 15 anos e 16 milhões de partos entre aquelas com 15- 19 anos (2). Nos países de baixa e média renda, 50,0% dessas gestações não são planejadas (3). A taxa gestacional brasileira é de 68,4 nascimentos para cada 1 mil adolescentes entre 15 e 19 anos, o que totaliza 364.734 nascimentos, dos quais 17.456 ocorrem entre meninas de 10-14 anos (4). Essas estatísticas ressaltam a urgência de compreender e abordar essa realidade, uma vez que adolescentes que vivenciam a maternidade apresentam maior prevalência de depressão e ansiedade (5).

A saúde mental das mães adolescentes é uma área crítica de investigação, devido às suas implicações para o bem-estar das jovens e o desenvolvimento de seus filhos (6). Estima-se que 14,0% dos adolescentes no mundo vivenciam transtornos mentais (7). Isso evidenciou a importância de entender como a gravidez na adolescência pode interagir com essas condições psicológicas. No Brasil, a gestação na adolescência relaciona-se a desafios psicológicos e sociais, como depressão, transtornos de conduta e hiperatividade, que podem impactar negativamente o desenvolvimento das mães e de seus filhos (6,8).

É fundamental reconhecer que a gravidez na adolescência é frequentemente estigmatizada e associada a comportamentos de risco, o que contribui para a visão simplista do fenômeno e de suas causas subjacentes (1,9). Esse estigma pode dificultar a identificação e a abordagem adequada dos problemas de saúde mental enfrentados por mães adolescentes. Isso sublinha a necessidade da abordagem compreensiva que considere não apenas os aspectos biológicos, mas também os contextos sociais, culturais e históricos que influenciam essa realidade (10,11).

Este artigo analisou a saúde mental das mães adolescentes brasileiras usuárias do Sistema Único de Saúde (SUS) ao longo de sua trajetória materna. Por meio de investigação abrangente e multidisciplinar, buscou-se contribuir para a melhor compreensão dos desafios enfrentados pelas adolescentes e identificar estratégias eficazes para promover sua saúde mental e o bem-estar de seus filhos.

## Métodos

### Delineamento do estudo

Tratou-se de um estudo multicêntrico com delineamento transversal. 

### Contexto

O estudo foi baseado na pesquisa “Vulnerabilidades da Gestação Precoce no Brasil: impactos na mãe adolescente e na criança”, realizada por meio do Programa de Apoio ao Desenvolvimento Institucional do SUS. A coleta de dados ocorreu entre agosto de 2022 e maio de 2023, através de entrevistas realizadas nas residências ou durante consultas hospitalares. O estudo seguiu as diretrizes e normas da Resolução nº 466/2012 do Conselho Nacional de Saúde (12).

### Participantes

Foram incluídas mães com idades entre 10 e 19 anos completos que realizaram o parto nas instituições selecionadas entre junho de 2021 e junho de 2022. Todas assinaram o registro do consentimento livre e esclarecido ou o registro de assentimento livre esclarecido, e eram fluentes em português, capazes de compreender os objetivos do estudo. As adolescentes que atendiam aos critérios de inclusão foram convidadas a participar por contato telefônico. A escolha de incluir mães com parto entre junho de 2021 e junho de 2022 visou evitar o período de restrições hospitalares devido à pandemia de covid-19, o que garantiu acompanhamento adequado e condições de bom estado físico e mental para o estudo.

### Variáveis

Para o desfecho de diagnóstico de depressão e ansiedade, foi criada a variável dicotômica a partir do questionário da Escala de Depressão, Ansiedade e Estresse (DASS-21); mães adolescentes com respostas diferentes de “normal” nos domínios de depressão e ansiedade foram classificadas como “sim”. Os modelos incluíram variáveis demográficas, escolaridade, emprego, apoio familiar, saúde geral, gestacional e mental como variáveis independentes. As variáveis a seguir foram utilizadas como covariáveis independentes.

- Demográfico: idade da mãe (anos); estado civil (solteira, casada, união estável); raça/cor da pele (branca, não branca); escolaridade (ensino fundamental, ensino médio, ensino superior); situação empregatícia (trabalhando, desempregada, nunca trabalhou); e idade do pai da criança (anos).
- Saúde da mãe adolescente antes da gestação (doenças diagnosticadas): depressão; ansiedade; transtornos psiquiátricos; doenças infecciosas; hematológicas; nefropatias; e desnutrição (sim, não).
- Depressão pós-parto: a mãe adolescente teve depressão pós-parto? (sim, não ).
- Interrupção dos estudos: parou de estudar durante a gravidez; e estava estudando quando o filho nasceu (sim, não).
- Apoio do pai da criança e familiar: recebe apoio emocional, apoio na rotina, apoio financeiro do pai da criança e apoio familiar (sim, não ).
- Apoio familiar: recebe apoio financeiro, apoio na rotina, apoio em saúde e apoio afetivo (sim, não).
- Envolvimento do pai da criança na gestação: como foi o envolvimento paterno durante a gestação? (bom, ótimo, regular, ruim ).
- Diagnósticos de saúde mental: algum médico ou profissional da saúde mental disse que a mãe adolescente tinha depressão ou ansiedade? (não, antes da gestação de 2021/2022, durante a gestação de 2021/2022 ).
- Fatores que influenciaram a gestação: engravidou sem querer; queria ser mãe; queria ter outro filho; queria construir uma família; queria sair da casa dos pais/responsáveis; queria casar; engravidou porque achava que seria mais respeitada após ser mãe; não sabia como evitar filhos; não tinha condições de comprar contraceptivos; casou cedo; não tinha outra opção; era um projeto de vida; o marido/companheiro queria ter filhos logo; queria desenvolver maior maturidade; o parceiro não queria usar camisinha; não sabia onde conseguir um anticoncepcional; foi vítima de abuso; e o contraceptivo falhou (verdadeiro, falso).
- Mudanças que ocorreram após a gestação: sua vida ficou mais difícil; sua vida ficou mais organizada; passou a ser mais respeitada; sua relação com o marido/companheiro melhorou; seu marido/companheiro lhe abandonou; foi rejeitada pela família; abandonou a escola/curso técnico/faculdade; teve vontade de estudar para dar um bom futuro para o bebê; sua vida melhorou porque formou o seu próprio lar; passou a ter um motivo para viver; foi o pior período da sua vida; passou a ficar melhor consigo mesma; casou; dificultou a obtenção e permanência no trabalho; perdeu a turma de amigos/escola; criou novas amizades ou se aproximou de mulheres que também são mães; e ficou mais difícil namorar (verdade, falso).

### Fonte de dados

Foram selecionados cinco hospitais de referência, localizados em capitais de cada uma das cinco regiões do Brasil, que apresentavam elevados indicadores de nascidos vivos de mães adolescentes (4). A seleção dos hospitais foi baseada no vínculo com o SUS e na certificação pela Iniciativa Hospital Amigo da Criança.

### Viés

As informações foram obtidas por meio de formulário estruturado elaborado pela equipe coordenadora do estudo. Entrevistadoras treinadas e certificadas conduziram as entrevistas para minimizar a possibilidade de viés. Realizaram-se o monitoramento das entrevistas e a conferência do banco de dados.

### Tamanho do estudo

A amostra necessária para o estudo foi de 583 participantes, levando em consideração que 26,3% das adolescentes poderiam apresentar ansiedade e depressão, além da possibilidade de que 10,0% das participantes não finalizassem o questionário. Para garantir que o estudo fosse capaz de detectar diferenças reais, utilizou-se o tamanho de amostra que assegurava alta probabilidade de identificar essas diferenças, com poder da amostra de 80% e intervalo de confiança de 95% (IC95%) (13). A amostragem foi não probabilística, incluindo as participantes de forma proporcional ao número de nascidos vivos de mães adolescentes por região do Brasil.

### Métodos estatísticos

A análise dos dados iniciou com a criação de banco de dados no Excel, no qual as informações foram codificadas e validadas por dupla digitação. As variáveis categóricas foram analisadas por frequências absolutas e relativas, e as contínuas por médias e desvios-padrão ou medianas e intervalos interquartis. O teste de Shapiro-Wilk avaliou a normalidade. Algumas variáveis foram recategorizadas para facilitar a interpretação, como a variável raça/cor da pele (branca e não branca) e escolaridade (ensino fundamental, médio, superior e não sabe), excluindo respostas “não querer responder” ou “não lembrar”.

A validação da escala DASS-21 para o português brasileiro foi realizada por tradução-retrotradução com 242 participantes, mostrando alta adequação ao modelo (teste Kaiser-Meyer-Olkin [KMO] de 0,949) e boas consistências internas (alfa de Cronbach: 0,92 para depressão, 0,90 para estresse e 0,86 para ansiedade). As correlações com as escalas de Beck e o Inventário de Sintomas de Stress de Lipp e a análise fatorial confirmaram a validade da DASS-21 para avaliar separadamente depressão, ansiedade e estresse (14).

A Escala DASS-21 é composta por três subescalas, cada uma com sete afirmativas, avaliando os estados emocionais na escala Likert de 0 a 3. Os níveis de intensidade (normal, leve, moderado, grave e extremamente grave) indicam a presença de sintomas (13). A análise fatorial confirmou a estrutura de três fatores distintos (14). “Normal” significa ausência de sintomas, e os resultados foram apresentados como presença ou ausência de sintomas e em níveis de intensidade. A pontuação total é a soma dos itens de cada subescala.

A associação entre as variáveis e a prevalência de ansiedade e depressão foi analisada por modelos de regressão de Poisson com variância robusta, estimando razões de prevalência e corrigindo problemas de superdispersão, o que garantiu IC95% precisos e confiáveis (15).

## Resultados

Participaram da pesquisa 583 mães adolescentes de todas as regiões do Brasil. Observaram-se as características socioeconômicas, educacionais, ocupacionais, o diagnóstico de depressão e ansiedade e o acesso ao tratamento de saúde mental (Tabela 1). A média de idade foi 18 anos (16-19 anos), e 83,7% se autodeclararam não brancas. A maioria era solteira (85,8%) e 69,4% participavam de programas sociais, com renda média de R$ 1.212,00 (R$ 880,00-R$ 1.900,00).

**Tabela 1:** Frequências absolutas e relativas (%) da caracterização socioeconômica, educacional, ocupacional e de saúde mental das mães adolescentes. Brasil, 2022-2023 (n=583)

Variável	**n** (%)
**Raça/cor da pele**	
Branca	95 (16,3)
Não branca	488 (83,7)
**Estado civil**	
Solteira	500 (85,8)
Casada	66 (11,3)
União estável	17 (2,9)
**Participa de programas sociais do governo**	403 (69,4)
**Estuda atualmente**	120 (20,5)
**Nível de escolaridade**	
Ensino fundamental (completo/incompleto)	199 (34,1)
Ensino médio (completo/incompleto)	371 (63,7)
Ensino superior (completo/incompleto)	13 (2,2)
**Motivos para não estar estudando**	
Gestação/ser mãe	261 (44,7)
Parou de estudar durante a gestação	300 (51,4)
**Situação ocupacional**	
Trabalhando	93 (15,9)
Desempregada	284 (48,7)
Nunca trabalhou	206 (35,3)
**Diagnóstico de depressão e** ansiedade	
Antes da gestação	88 (15.0)
Durante a gestação	44 (7,5)
Frequenta consultas de saúde mental	21 (15,9)
**Motivos para não realizar acompanhamento de saúde mental**	
Não conseguiu acesso ao suporte psicológico no Sistema Único de Saúde	26 (29,5)
Não tem interesse	23 (26,1)
Não foi estimulada a manter o acompanhamento	22 (25,0)
**Condição DASS**-21^a^	
Estresse	325 (55,7)
Ansiedade	372 (63,8)
Depressão	361 (61,9)

^a^Escala de depressão, ansiedade e estresse.

**Tabela 2:** Razão de prevalência (RP) e intervalo de confiança de 95% (IC95%) relacionados à ansiedade e à depressão das mães adolescentes. Brasil, 2022-2023 (n=583)

Variável	RP (IC95%)^c^	p-valor	RP (IC95%)^c^	p-valor
Modelo 1^a^ – demográfico^b^	Modelo bruto		Modelo ajustado	
**Idade da mãe** (anos)	0,96 (0,92; -1,00)	0,031	0,97 (0,94; 1,00)	0,094
**Estado civil**				
Solteira	1,00	-	1,00	-
Casada	0,96 (0,81; 1,14)	0,621	0,95 (0,80; 1,13)	0,565
União estável	1,25 (1,04; 1,51)	0,015	1,23 (1,03; 1,46)	0,024
**Raça/cor da pele**				
Branca	1,00	-	-	-
Não branca	0,92 (0,82; 1,04)	0,206	-	-
Escolaridade				
Fundamental (completo/incompleto)	1,00	-	-	-
Médio (completo/incompleto)	1,05 (0,93; 1,18)	0,435	-	-
Superior (completo/incompleto)	0,79 (0,47; 1,32)	0,367	-	-
**Situação empregatícia**				
Trabalhando	1,00	-	-	-
Desempregada	1,01 (0,87; 1,17)	0,875	-	-
Nunca trabalhou	0,91 (0,78; 1,07)	0,252	-	-
**Idade do pai da criança**	1,00 (0,99; 1,01)	0,665	-	-
**Escolaridade do pai da criança**				
Fundamental incompleto	1,00	-	-	-
Médio (completo/incompleto)	0,93 (0,83; 1,05)	0,229	-	
Superior (completo/incompleto)	0,97 (0,73; 130)	0,852		
Não sabe	0,90 (0,76; 1,07)	0.227	-	-
Modelo 2^a^ – saúde da mãe adolescente antes da gestação^b^	Modelo bruto		Modelo ajustado	
Depressão				
Sim	1,00	-	1,00	-
Não	0,84 (0,78; 0,92)	<0,001	0,85 (0,78; 0,92)	<0,001
**Ansiedade**				
Sim	1,00	-	1,00	-
Não	0,75 (0,68; 0,82)	<0,001	0,75 (0,68; 0,82)	<0,001
**Transtorno psiquiátricos**				
Sim	1,00	-	-	-
Não	1,04 (0,99; 1,10)	0,103	-	-
Infecciosas				
Sim	1,00	-	-	-
Não	0,93 (0,69; 1,26)	0,653	-	-
Hematológicas				
Sim	1,00	-	-	-
Não	0,89 (0,69; 1,15)	0,382	-	-
Nefropatias				
Sim	1,00	-	-	-
Não	1,51 (0,63; 3,63)	0,356	-	-
Desnutrição				
Sim	1,00	-	-	-
Não	1,02 (0,76; 1,37)	0,897	-	-
Modelo 3^a^ – diagnóstico de saúde mental pós-parto^b^	Modelo bruto		Modelo ajustado	
**Depressão pós**-parto				
Sim	1.00	-	-	-
Não	0,76 (0,69; 0,83)	<0,001	-	-

^a^Os modelos apresentados nesta tabela têm como variável resposta “Diagnóstico de depressão/ansiedade (DASS-21)”; ^b^Os modelos 1, 2 e 3 incluem diferentes variáveis independentes relacionadas às informações demográficas, aos diagnósticos antes da gestação e ao diagnóstico de saúde mental pós-parto; ^c^O efeito de cada variável explicativa foi avaliado por meio de RP com IC95%.

A média de idade dos pais foi 22 anos (20-25 anos). A porcentagem de mães que estavam estudando foi 20,5% sendo o ensino médio completo ou incompleto o mais comum (63,7%). Entre as que não estudavam 44,7% citaram a maternidade como motivo e 51,4% interromperam os estudos devido à gestação. A maioria estava desempregada (48,7%), com 15,9% exercendo alguma atividade.

Foram diagnosticadas com depressão e ansiedade 132 adolescentes (22,6%); 88 (15,0%) foram diagnosticadas antes da gestação, e 44 (7,5%), durante. Entre as diagnosticadas, 21 (15,9%) faziam acompanhamento psicológico. As que receberam o diagnóstico antes da gestação mencionaram dificuldades de acesso ao suporte psicológico pelo SUS (26 [29,5%]), desinteresse (23 [26,1%]) ou falta de estímulo (22 [25,0%]) (Tabela 1). A prevalência de sintomas de depressão, ansiedade e estresse foi 61,9%, 63,8% e 55,7%. A prevalência de depressão e ansiedade foi 25,0% maior entre adolescentes em união estável. As que não tinham diagnóstico prévio de depressão apresentaram 15,0% menos sintomas. Houve associação significativa entre a saúde mental e o diagnóstico de depressão pós-parto (p-valor<0,001). Adolescentes sem depressão pós-parto apresentaram 24,0% menos prevalência de depressão ou ansiedade.

As adolescentes responderam afirmativas com “verdadeiro” ou “falso” (Tabela Suplementar 1). As que afirmaram que a vida ficou mais difícil após a gestação apresentaram 22,0% mais prevalência de depressão/ansiedade (p-valor 0,002). Aquelas que perderam amigos/escola tiveram 23,0% mais prevalência de resultados positivos no DASS-21 (p-valor 0,031). As adolescentes que se sentiram mais respeitadas apresentaram 10,0% menos prevalência de depressão/ansiedade (p-valor<0,001).

A análise das variáveis investigadas mostrou que a falta de apoio familiar aumentou a prevalência de sintomas (p<0,001). Adolescentes sem apoio familiar apresentaram 32,0% mais prevalência de depressão/ansiedade. As que sentiram falta de apoio na rotina com o bebê apresentaram 23,0% mais prevalência (Tabela 3). Adolescentes que consideraram o envolvimento do pai durante a gestação como ótimo apresentaram 15,0% menos prevalência de depressão/ansiedade (p-valor 0,014). Aquelas com diagnóstico de depressão antes ou durante a gestação apresentaram 36,0% mais prevalência de sintomas no momento da entrevista (Tabela 3).

**Tabela 3:** Razão de prevalência (RP) e intervalo de confiança de 95% (IC95%) relacionados à ansiedade, à depressão e ao estresse antes e durante a gravidez das mães adolescentes. Brasil, 2022-2023 (n=583)

Variável	RP (IC95%)^c^	p-valor	RP (IC95%)^c^	p-valor
Modelo 4^a^ – interrupção dos estudos^b^	Modelo bruto		Modelo ajustado	
**Parou de estudar durante a gravidez**				
Não	1,00	-	-	-
Sim	1,09 (0,98; 1,21)	0,096	-	-
**Estava estudando quando o filho nasceu**				
Não	1,00	-	-	-
Sim	0,96 (0,86; 1,07)	0,431	-	-
Modelo 5^a^ – apoio familiar e do pai da criança^b^	Modelo bruto		Modelo ajustado	
**Recebe apoio emocional**				
Sim	1,00	-	-	-
Não	1,13 (0,96; 1,32)	0,135	-	-
**Recebe apoio na rotina**				
Sim	1,00	-	-	-
Não	1,09 (0,94; 1,26)	0,245	-	-
**Recebe apoio financeiro**				
Sim	1,00	-	-	-
Não	0,89 (0,77; 1,03)	0,114	-	-
**Recebe apoio familiar**				
Sim	1,00	-	-	-
Não	1,32 (1,19; 1,46)	<0,001	-	-
Modelo 6^a^ – apoio da família^b^	Modelo bruto		Modelo ajustado	
**Recebe apoio financeiro**				
Sim	1,00	-	-	-
Não	1,04 (0,92; 1,18)	0,535	-	-
**Recebe apoio na rotina**				
Sim	1,00	-	1,00	-
Não	1,21 (1,07; 1,37)	0,003	1,23 (1,11; 1,37)	<0,001
**Recebe apoio na saúde**				
Sim	1,00	-	-	-
Não	1,01 (0,89; 1,15)	0,911	-	-
**Recebe apoio afetivo**				
Sim	1,00	-	1,00	-
Não	1,13 (1,00; 1,29)	0,059	1,14 (1,00; 1,30)	0,055
Modelo 7^a^ – envolvimento paterno na gestação^b^	Modelo bruto		Modelo ajustado	
**Envolvimento do pai da criança na gestação**				
Bom	1,00	-	-	-
Ótimo	0,85 (0,74; 0,97)	0,014	-	-
Regular	1,08 (0,94; 1,24)	0,296	-	-
Ruim	0,97 (0,82; 1,14)	0,690	-	-
Modelo 8^a^ – diagnóstico de saúde mental^b^	Modelo bruto		Modelo ajustado	
**Diagnósticos de depressão**/ansiedade				
Não	1,00	-	-	-
Antes da gestação de 2021/2022	1,36 (1,24; 1,49)	<0,001	-	-
Durante a gestação de 2021/2022	1,36 (1,22; 1,53)	<0,001	-	-

^a^Os modelos apresentados nesta tabela têm como variável resposta “Diagnóstico de depressão/ansiedade (DASS-21)”; ^b^Os modelos 4, 5, 6, 7 e 8 incluem diferentes variáveis independentes relacionadas às informações sobre interrupção dos estudos, ao apoio do pai da criança, ao apoio da família, ao envolvimento paterno durante a gestação e ao diagnóstico de saúde mental; ^c^O efeito de cada variável explicativa foi avaliado por meio de RP com IC95%.

## Discussão

Os achados desta pesquisa revelaram a realidade alarmante da saúde mental entre mães adolescentes no Brasil e evidenciou a relação entre as condições socioeconômicas e os sintomas de depressão e ansiedade. A maioria das participantes tinha 18 anos, era solteira e vivia em situação socioeconômica vulnerável, com alta participação em programas sociais. A baixa escolaridade e a interrupção dos estudos, muitas vezes devido à maternidade, agravaram a saúde mental. A prevalência de sintomas foi maior entre aquelas sem apoio familiar e com dificuldades de acesso a serviços de saúde mental. O apoio paterno durante a gestação foi fator protetor, o que indicou que relações familiares positivas podem melhorar a saúde emocional. Esses resultados reforçaram a necessidade de intervenções que abordem tanto o apoio psicológico quanto as condições sociais dessa população.

Este estudo teve limitações. A falta de estratificação das adolescentes em duas faixas etárias (10-14 anos e 15-19 anos) limitou a análise, especialmente pela inadequação da amostra de mães com menos de 14 anos. Apesar de a amostra incluir adolescentes de diversas regiões, todas as participantes foram atendidas no SUS. o que manteve a homogeneidade. A falta de avaliação regionalizada limitou a compreensão de variações nos contextos das regiões brasileiras. As entrevistas foram autorrelatadas, podendo introduzir viés de memória. A maioria das gestações ocorreu durante a emergência sanitária da covid-19, o que pôde ter impactado o acesso aos serviços de saúde e o isolamento social.

Os dados revelaram que 361 adolescentes (61,9%) apresentavam algum nível de depressão, 372 (63,8%) manifestavam ansiedade e 325 (55,7%) exibiam estresse. Esses resultados foram preocupantes e sugeriram prevalência significativamente maior de problemas de saúde mental entre adolescentes mães quando comparados à análise realizada com estudantes brasileiros, que encontrou 11,7% de casos de depressão, 10,2% de ansiedade e 11,3% de estresse, utilizando a mesma métrica da DASS-21 (16). Embora a análise citada não tenha sido realizada com adolescentes grávidas ou com partos recentes, evidenciaram-se diferenças no impacto da gravidez sobre a saúde mental, o que refletiu as pressões adicionais que a maternidade impõe.

Os níveis de ansiedade, depressão e estresse associaram-se ao diagnóstico de depressão pós-parto (p-valor<0,001), ao aumento das dificuldades após o parto (p-valor 0,002) e à perda de amigos da escola (p-valor<0,001). Essas associações indicaram que as dificuldades pós-parto puderam estar ligadas ao aumento das responsabilidades da maternidade, enquanto a perda de amigos refletiu a incompatibilidade entre a rotina anterior e as novas exigências (17,18). A rede de apoio social foi crucial nesse período, e sua ausência pôde intensificar o isolamento e a depressão (19). 

No Nordeste brasileiro, 56,5% das adolescentes tinham risco mínimo de depressão, contudo esta pesquisa foi realizada com menor amostra e durante a gestação, o que pode explicar as diferenças (20). Internacionalmente, até 29,0% das mães adolescentes apresentavam depressão, o que reforçou a necessidade de atenção especializada (21). Comparando a saúde mental de adolescentes grávidas e não grávidas, 24,6% das grávidas mostravam sinais de problemas mentais, enquanto 27,3% das não grávidas tinham problemas (5). As diferenças nos instrumentos de avaliação puderam contribuir para os resultados contrastantes. 

Observou-se que as adolescentes que se sentiram mais respeitadas após a gestação apresentaram prevalência 10,0% menor de níveis positivos de ansiedade e depressão (p-valor 0,031), o que demonstrou que a gravidez na adolescência também pode contribuir, de forma protetiva, para a saúde mental materna. Esse dado se relaciona com as percepções de autoeficácia e autoestima, na quais a visão de valor e respeito se demonstram protetivos para a depressão nos primeiros meses após a gravidez (19).

Entre as 583 adolescentes participantes desta pesquisa, 132 (22,6%) foram diagnosticadas com ansiedade ou depressão em algum momento da vida. Esse dado é superior ao encontrado em Pernambuco, onde 15,6% das participantes apresentaram diagnóstico de transtorno mental antes da gestação (22). Este estudo demonstrou que o diagnóstico anterior (p-valor<0,001) ou durante a gestação (p-valor<0,001) possuiu associação importante com os níveis de depressão, ansiedade e estresse no momento da entrevista. O histórico de transtornos mentais antes da gestação foi fator de risco significativo para o desenvolvimento de novos sintomas durante a gravidez, afetando a saúde mental de mulheres de diversas faixas etárias (23). Para adolescentes, essa preocupação se tornou ainda mais alarmante, dada a vulnerabilidade emocional característica dessa fase do desenvolvimento (19).

Apesar de a significativa proporção de adolescentes apresentar indicativos de ansiedade ou depressão no momento da entrevista, apenas 21 (15,9%) relataram frequentar consultas de saúde mental. As que tiveram diagnóstico há mais tempo (antes da gestação) mencionaram dificuldades de acesso ao suporte psicológico pelo SUS (26 [29,5%]), desinteresse (23 [26,1%]) ou ausência de estímulo para manter o acompanhamento (22 [25,0%]). Parte significativa das mães com ansiedade (48,0%) e depressão (70,0%) continuou enfrentando sofrimento psicológico por não receber orientação e apoio adequados (24). Muitas não buscaram ajuda devido aos estigmas sociais ou à dificuldade de acesso a recursos, resultando em baixa adesão ao tratamento e ao acompanhamento de sua condição (25,26). 

A normalização do sofrimento psicológico nas mães adolescentes é um desafio que precisa ser enfrentado com estratégias de saúde pública que priorizem a sensibilização e a educação em saúde mental. A escassez de recursos financeiros contribui para o acesso limitado à saúde mental (27,28). Esse cenário é alarmante, pois mães que apresentam sofrimento psíquico antes do nascimento do bebê têm alta probabilidade de continuar a apresentar ou agravar seus sintomas após o parto (24,28). A atenção profissional e o encaminhamento adequado ao atendimento especializado são essenciais para a melhoria da saúde mental materna (29).

No cenário brasileiro, meninas que não têm apoio do parceiro durante a gestação apresentaram 1,4 vez mais chances de desenvolver transtornos mentais (30). Adicionalmente, 57,0% das mães adolescentes sem apoio social e familiar durante ou após a gravidez apresentaram sintomas depressivos quatro anos após o parto (31). Os dados deste estudo corroboram a percepção de que o suporte social é fundamental para que as adolescentes não se sintam sobrecarregadas com as novas responsabilidades, funcionando como forma de proteção da saúde mental (24,31). O envolvimento do pai da criança no cuidado pode contribuir nos desfechos relacionados à saúde mental das adolescentes e proteger o bebê de riscos socioemocionais envolvidos nos casos de depressão materna (21).

Este estudo destacou os índices preocupantes de depressão, ansiedade e estresse entre mães adolescentes atendidas no SUS. As associações com apoio social, planejamento familiar e diagnóstico clínico ofereceram análises importantes para intervenções eficazes. É necessário planejar políticas de saúde que previnam agravos e promovam a saúde mental, incluindo suporte emocional e tratamento adequado. A falta de acesso à saúde mental no SUS é uma preocupação urgente que exige reavaliação. Ações devem ser implementadas de forma integrada, envolvendo os serviços de saúde, a comunidade e a família, para garantir que as mães adolescentes recebam o suporte necessário durante esse período crítico de suas vidas.

## Data Availability

O banco de dados e os códigos de análise utilizados na pesquisa estão disponíveis em: https://drive.google.com/drive/folders/1ER03g1jnm9pAyZYmwBmU5gxzHBaf7fBu?usp=drive_link.

## Material suplementar

Tabela suplementar 1.
